# Contralateral Astrocyte Response to Acute Optic Nerve Damage Is Mitigated by PANX1 Channel Activity

**DOI:** 10.3390/ijms242115641

**Published:** 2023-10-27

**Authors:** Jasmine A. Wurl, Caitlin E. Mac Nair, Joel A. Dietz, Valery I. Shestopalov, Robert W. Nickells

**Affiliations:** 1 Department of Ophthalmology and Visual Sciences, University of Wisconsin-Madison, Madison, WI 53706, USA; jwurl@wisc.edu (J.A.W.); cemacnair@gmail.com (C.E.M.N.); jadietz@wisc.edu (J.A.D.); 2 Bascom Palmer Eye Institute, University of Miami School of Medicine, Miami, FL 33136, USA; vshestopalov@med.miami.edu; 3 McPherson Eye Research Institute, University of Wisconsin-Madison, Madison, WI 53705, USA

**Keywords:** astrocyte, Müller cell, unilateral optic nerve damage, contralateral retina, PANX1 hemichannels

## Abstract

Glial reactivity is considered a hallmark of damage-induced innate immune responses in the central nervous system. In the visual system, unilateral optic nerve damage elicits dramatic glial reactivity in the retina directly affected by the lesion and a similar, albeit more modest, effect in the contralateral eye. Evaluation of astrocyte changes in a mouse model of optic nerve crush indicates that astrocyte reactivity, as a function of retinal coverage and cellular hypertrophy, occurs within both the experimental and contralateral retinas, although the hypertrophic response of the astrocytes in the contralateral eyes is delayed for at least 24 h. Evaluation of astrocytic reactivity as a function of *Gfap* expression indicates a similar, muted but significant, response in contralateral eyes. This constrained glial response is completely negated by conditional knock out of *Panx1* in both astrocytes and Müller cells. Further studies are required to identify if this is an autocrine or a paracrine suppression of astroglial reactivity.

## 1. Introduction

Reactive gliosis is a well-established phenomenon associated with neurodegenerative conditions [1]. The principal innate immune signaling cells in the central nervous system are microglia. These cells are dynamic sentinels of their environment with relatively small cell bodies and numerous thin processes [2]. In response to damage, the microglia react by retracting and thickening processes, initiating migration to the wound site, and then finally re-extending processes to surveil the surrounding area [3]. They also provide antigen presenting sites and synthesize inflammatory cytokines to elicit further immune cell integration [4,5,6]. Astrocytes normally function to maintain homeostatic balance of the extracellular environment, provide energy sources to adjacent neurons, and modulate synapse formation and function [7]. These cells form static or fixed domains in neural tissues and are characterized by small cell bodies with several long processes that extend to blood vessels, neurons, and other astrocytes. Upon tissue damage, astrocytes become reactive and change their transcriptome [8,9]. A principal marker for reactive astrocytes used by investigators is monitoring for an increase in intermediate filament expression including vimentin and glial fibrillary acidic protein (GFAP). Morphologically, they exhibit signs of hypertrophy, including an increase in the complexity, number, and thickening of processes while maintaining the coverage of their domain [10]. Importantly, reactive gliosis for both microglia and astrocytes are not characterized by a simple transformation from a non-reactive to a reactive phenotype, but rather represents a wide range of heterogenous states as these cells adapt to their environment [3,11,12].

In the retina, reactive gliosis is characteristic of both acute (crush or axotomy) and chronic (ocular hypertension leading to glaucoma) damage of the optic nerve [13,14,15,16,17,18,19]. Microglia populate three retinal layers including the nerve fiber layer and both plexiform layers, while astrocytes are restricted to the nerve fiber layer. In addition, the retina contains specialized macroglia called Müller cells which have cell bodies contained in the inner nuclear layer and extend processes from the inner to the outer limiting membranes. The gliotic response is dramatic in retinas of eyes directly exposed to optic nerve damage but can also be detected in contralateral retinas [14,20,21,22,23,24]. The precise mechanism of communication between injured and contralateral retinas is not fully elucidated, but recent studies indicate that astrocytes form a syncytium through up-regulation of connexin-43-containing gap junctions [25] that allow for a transfer of materials from non-injury to injury sites [26,27] and presumably in the reverse direction as well. Several other models have been proposed including damage to retino-retinal projecting RGCs, signals emanating from the optic tract after deafferentation, and systemic effects associated with monocyte invasion [28].

The signaling pathways associated with the responses of the retinal glial populations are partially elucidated. In acute optic nerve damage, both the microglial and macroglial (astrocytes and Müller cells) responses are dramatically attenuated in conditions that prevent retinal ganglion cell (RGC) death [19,29], suggesting that this event is the primary signal leading to reactive gliosis. Retinal astrocytes are induced into a reactive “A1” phenotype by the presence of factors released by reactive microglia, including IL-1β, TNFα, and C1QA [30]. Under chronic damaging conditions, reactive astrocytes become neurotoxic and secrete molecules that exacerbate the death of damaged RGCs [31]. By default, these observations suggest a direct link between early RGC death and the induction of microglial reactivity. While this link has not yet been fully established in pathology, during retinal development, dying neurons drive microglial remodeling states [32]. Hypothetically, it seems likely that an initial period of RGC loss may be associated with the release of damage-associate molecular pattern molecules (DAMPs) that stimulate pattern recognition receptors, such as toll-like receptors (TLRs), on both microglia and macroglia [33,34,35]. Consistent with this, TLR function has been linked to RGC pathology in both acute injury models [36,37,38] and human normal tension glaucoma [39,40].

While experimental evidence indicates that astrocyte reactivity is induced by IL-1β, TNFα, and C1QA secreted by microglia, their reactivity is also modulated by purinergic signaling pathways. A principal signaling ligand in this pathway is ATP, which activates puringeric receptors in the P2X family. In individual cells, P2X family receptors are closely associated with PANX hemichannels which are the primary channels that allow for cells to release ATP to the extracellular environment. Because of this close apposition, ATP can immediately bind to the near P2X receptor, thereby creating an autocrine signaling mechanism [41,42,43,44,45]. A P2X channel agonist, BzATP, is able to spontaneously induce *Gfap* expression in retinal macroglia, while the antagonist oxATP, injected either before or after optic nerve injury, can suppress the injury-induced expression of this gene [19], principally in astrocytes. Other studies, however, suggest that the intrinsic interaction of PANX1 and P2X7 receptor is more complex, with PANX1 acting to modulate P2X7 activities both positively and negatively [42,46]. In assessing the role of ATP release from RGCs after optic nerve injury, we explored the conditional deletion of *Panx1* from these cells and Müller cells. The ablation of *Panx1* had no effect in limiting the activation of *Gfap* gene expression but did impact the ability of experimental retinas to turn this response off over time [19]. Here, we examine further the effect of conditional deletion of PANX1 channels in retinal macroglia in the acute optic nerve crush model. Reactive astrogliosis as a function of cell morphology and retinal coverage was similar between the experimental and contralateral eyes, although the response in the contralateral retina was delayed by several days and *Gfap* expression was only modestly up-regulated. Surprisingly, conditional knock-out of *Panx1* results in a hyperactivation of *Gfap* expression that is restricted to astrocytes in the contralateral retina. These results suggest a negative feed-back mechanism for the glial response utilizing PANX1 channels, which could be either autocrine, or paracrine, or both. Since suppression of gliotic activity is a major challenge for successful RGC transplantation and/or recovery from damage, understanding how this is controlled in the contralateral retina may provide key insights for future therapeutic interventions [47].

## 2. Results

### 2.1. Astrocyte Coverage and Morphology Changes after Optic Nerve Crush

**Figure 1:**
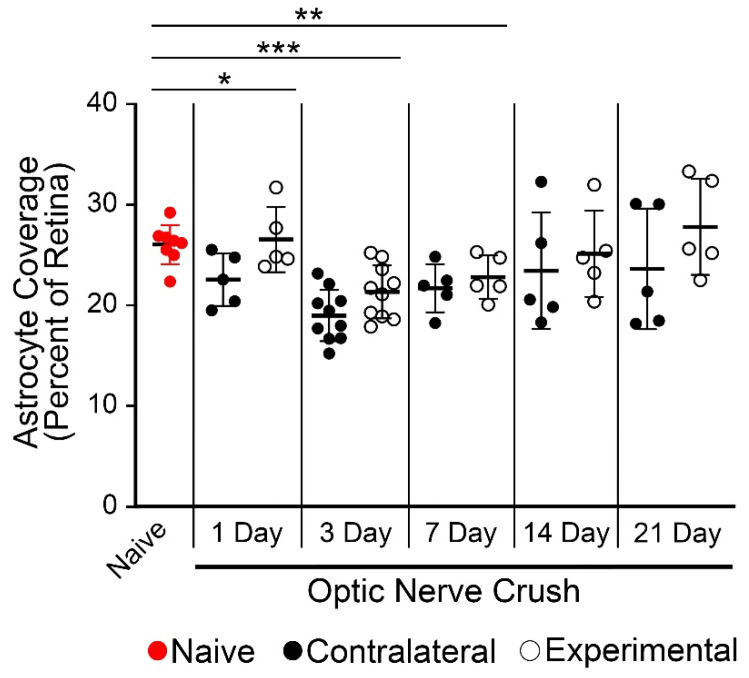
Retinal coverage by astrocytes after optic nerve crush. Scatter plot showing mean and standard deviation of the percentage of retinal coverage by astrocytes. Each point represents the average coverage of an individual retina. There was no significant difference in the percent coverage between experimental and contralateral retinas at any time point after crush surgery. In group comparisons, the retinas from mice with unilateral optic nerve injury showed a decrease in coverage at 1, 3, and 7 days after crush compared to naïve retinas (* *p* = 0.043, ** *p* = 0.0058, *** *p* < 0.0001, ANOVA). Notably, the observed group decrease at 1 day appears to be driven by changes in the contralateral eyes, which were significantly different than naïve eyes (*p* = 0.018, *t*-test), while experimental eyes were not significantly different (*p* = 0.734, *t*-test). At 14 and 21 days after crush, both experimental and contralateral eyes exhibited a large variance in the percent coverage, but there was no significant difference between these eyes compared to naïve retinas in group comparisons.

**Figure 2:**
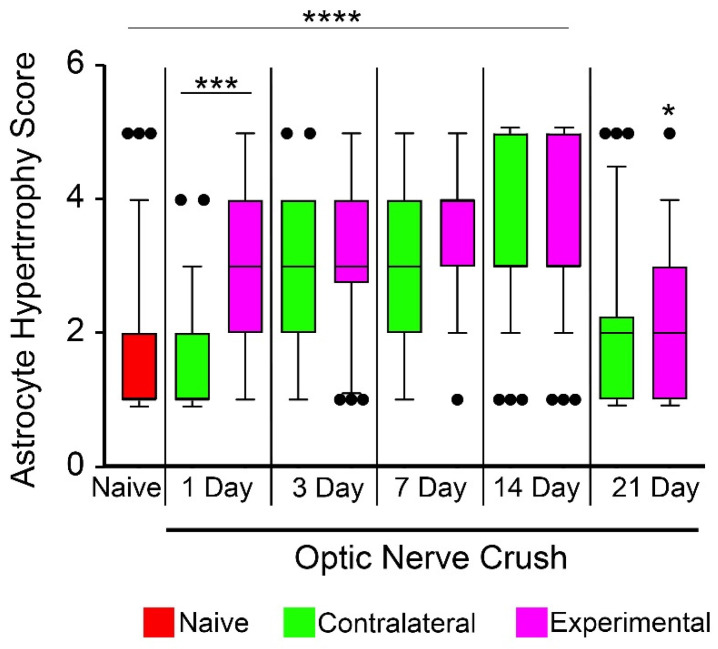
Astrocyte hypertrophy scoring after unilateral optic nerve crush injury. Box and whisker plots of astrocyte scores from retinas at increasing times after optic nerve crush compared to naïve retinas. Outlier data are graphed as individual points. Each plot represents scores from fields imaged from a minimum of 3 retinas (minimum of 30 cells scored). One day after crush surgery, experimental retinas exhibited a significant increase in the astrocyte hypertrophy score compared to both naïve and contralateral retinas (*** *p* < 0.0001, individual *t*-tests). By 3 days, and extending over 7 and 14 days, both contralateral and experimental retinas exhibited hypertrophic changes compared to naïve retinas (**** *p* < 0.0001, ANOVA). On days 3–21, the hypertrophy score was not significantly different between the contralateral and experimental retinas, although experimental eyes still exhibited a modest difference in score compared to naïve samples (* *p* = 0.022).

The reactivity of astrocytes was assessed using two criteria, astrocyte coverage of the retina based on GFAP immunoreactivity and astrocyte morphology changes. Astrocyte coverage between the experimental and contralateral retinas was not significantly different between eyes at any time point after optic nerve crush (Figure 1). Compared to naïve retinas, however, astrocyte coverage of the retina decreased significantly in both the experimental and contralateral eyes at 1 day after surgery (*p* = 0.043, ANOVA) persisting through 3 and 7 days (*p* < 0.0001 and 0.0058, respectively, ANOVA). At 14 and 21 days, retinal coverage in both groups was not significantly different from naïve retinas. A decrease in coverage has been documented by other groups assessing this metric in aging mice and rat models of glaucoma [14,48]. Evaluation of hypertrophic changes in GFAP-labeled astrocytes was assessed by scoring cell shape changes using a 1–5 scoring system (see Methods) with three masked observers (Figure 2). Cells in the experimental retina exhibited a significant increase in hypertrophy within 1 day after optic nerve crush (*p* < 0.0001, *t*-test, relative to both naïve and contralateral retinas), although these retinas exhibited broad heterogeneity in the cell shapes observed. By 3 days, cells in the contralateral retinas also exhibited significant hypertrophy that continued to escalate up to 14 days after optic nerve crush. At 3, 7, and 14 days there was a significant difference in the hypertrophy score of both the experimental and contralateral retinas compared to naïve retinas (*p* < 0.0001, ANOVA) and these were not significantly different from each other. At 21 days post-surgery, both groups of retinas exhibited a reduced hypertrophy score that approached the score measured in naïve retinas (*p* = 0.022, *t*-test, for experimental retinas; *p* = 0.256 for contralateral retinas).

### 2.2. Macroglial PANX1 Regulates Gfap Gene Expression in the Contralateral Retina after Unilateral Optic Nerve Damage

**Figure 3:**
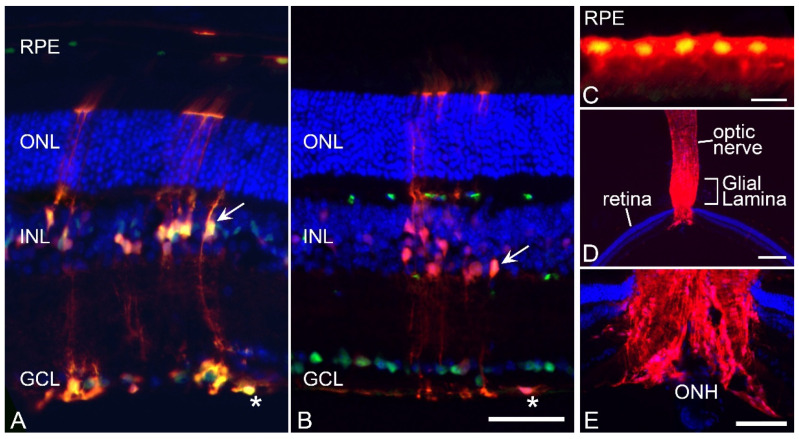
Validation of CRE activity in retinal macroglia. To confirm that *Gfap*-Cre transgenic mice successfully modified gene expression in target cells of the retina, this line was crossed with *Rosa26*-(LoxP)-tdTomato reporter mice and analyzed in frozen sections. (**A**) Section showing tdTomato fluorescence counterstained with an antibody against SOX9 which labels both astrocytes (asterisk) and Müller cells (arrow) and subsets of retinal pigmented epithelium (RPE) cells. The tdTomato transgene is expressed in the entire astrocyte population and a subset of Müller cells. (**B**) Section showing tdTomato fluorescence counterstained with an antibody against BRN3A, which stains retinal ganglion cells (RGCs). RGCs are negative for tdTomato expression and expression in the astrocytes (asterisk) and Müller cell (arrow) end feet are clearly visible adjacent to the ganglion cell layer (GCL). Scale bar (**A**,**B**) = 100 µm. (**C**) Higher magnification of a section of the RPE layer showing a cluster of tdTomato-expressing cells (counterstained with anti-SOX9). Scale bar = 20 µm. (**D**,**E**) low and higher magnification images of the optic nerve and optic nerve head (ONH) showing robust staining in the glial lamina. Scale bar (**D**) = 750 µm. Scale bar (**E**) = 300 µm. All sections were counter-stained with DAPI. Outer nuclear layer (ONL). Inner nuclear layer (INL).

To first assess the effect of PANX1 activity in the contralateral retina we used *Gfap*-Cre-expressing mice to delete *Panx1* in macroglia. Initially, we crossed the Cre-expressing line with the *Rosa26*-(LoxP)-tdTomato reporter strain to confirm expression of Cre in macroglia. Histologic evaluation of tdTomato expression in macroglia was confirmed by counter-staining frozen sections of naïve double transgenic mice with an antibody against SOX9, which is expressed in both astrocytes and Müller glia [49,50,51] and retinal pigmented epithelial cells [52]. Figure 3 shows robust tdTomato expression in a majority of astrocytes in the nerve fiber layer and subsets of Müller cells, suggesting at least low levels of *Gfap* gene expression in these cells, even though GFAP levels are not readily apparent in Müller cells until after injury [18,53,54,55]. Notably, tdTomato was not expressed in cells comprising the ganglion cell layer when counter-stained with the RGC marker BRN3A. Robust staining was also detected throughout the optic nerve. Surprisingly, subsets of retinal pigmented epithelial cells were also positive for tdTomato.

**Figure 4:**
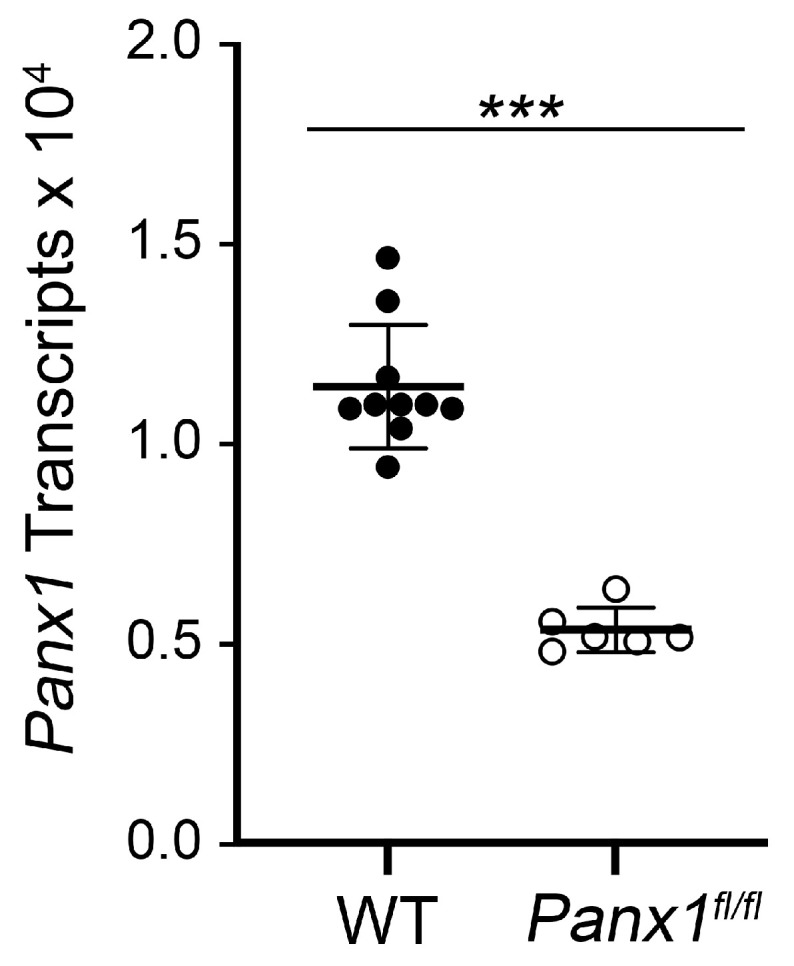
Quantitative RT-PCR of retinal *Panx1* mRNA levels. Scatter plot of the absolute levels of Panx1 transcripts in retinas of naïve wild-type (WT) or *Panx1^fl/fl^* (macroglia) mice. Each data point represents the message level in a single retina, normalized to the amount of mRNA of *S16* ribosomal protein. *Panx1^fl/fl^* mice exhibit a greater than 2-fold decrease in mRNA abundance (*** *p* < 0.0001, *t*-test).

**Figure 5:**
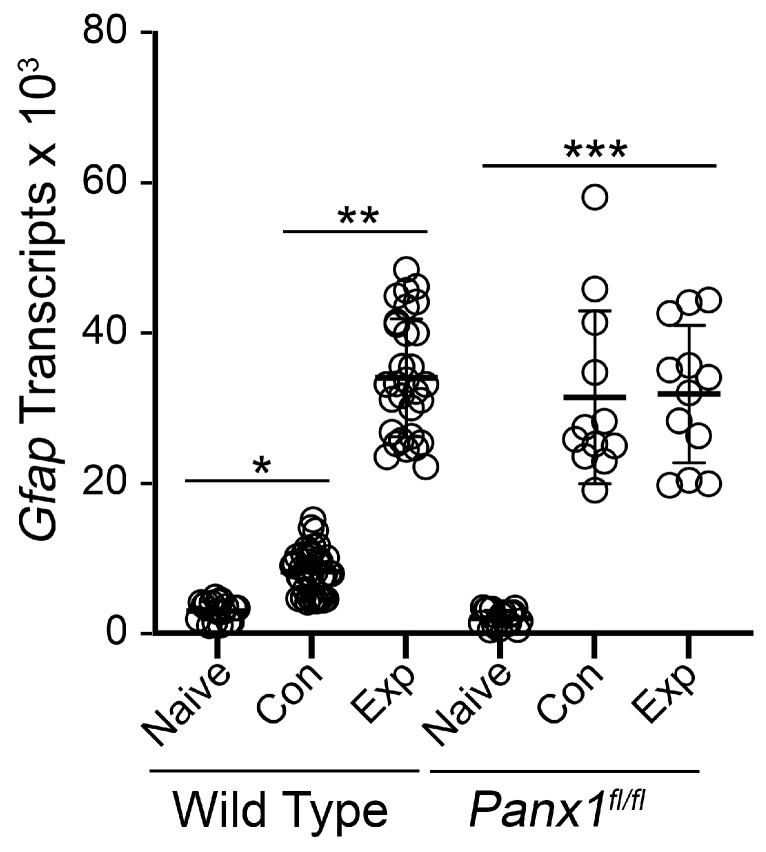
Quantitative analysis of retinal *Gfap* transcript abundance after optic nerve injury. Scatter plot of qPCR evaluation of the absolute abundance of *Gfap* transcripts in retinas of wild-type and *Panx1^fl^*^/*fl*^ mice comparing levels present in naïve retinas compared to both the contralateral and experimental retinas 7 days after optic nerve crush surgery. Each point represents an individual retina. Wild-type mice exhibit a significant increase in *Gfap* mRNA in both the contralateral retinas (* *p* < 0.0001) and experimental retinas (*p* < 0.0001), although levels were significantly higher in the experimental eyes relative to contralateral eyes (** *p* < 0.0001). Similarly, in mice with *Panx1* deleted in macroglia, both contralateral and experimental retinas exhibited a significant increase in *Gfap* mRNA levels relative to naïve mice (*** *p* < 0.0001) but at levels that were statistically equivalent to each other and to experimental retinas in wild-type mice (*p* = 0.866).

*Gfap*-Cre-expressing mice were then crossed with mice carrying a floxed allele for *Panx1*. To validate the suppression of *Panx1* gene expression in double transgenic mice, we conducted qPCR for absolute *Panx1* transcript abundance in retinas of naïve animals. Figure 4 shows that Cre-expressing mice exhibited an average 2.14-fold reduction in *Panx1* mRNA abundance compared to non-Cre-expressing mice. We then evaluated *Gfap* mRNA levels at 7 days after optic nerve crush surgery in both non-Cre and Cre-expressing mice (Figure 5). A seven day endpoint was evaluated because this represents the time when astrocyte *Gfap* expression and hypertrophy changes are still increasing in experimental eyes [19] (Figure 2). In non-Cre-expressing mice, both the contralateral and experimental retinas exhibited a significant increase in *Gfap* mRNA levels relative to naïve retinas, although the increase in *Gfap* expression was approximately 4-fold greater in the experimental eye. In Cre-expressing *Panx1^fl/fl^* mice, *Gfap* mRNA levels were actually lower (32% reduction in mean value) in the naïve retina compared to naïve non-Cre-expressing animals (*p* = 0.018, *t*-test). At 7 days after optic nerve crush, however, both the contralateral and experimental retinas exhibited a significant increase in *Gfap* mRNA levels that was statistically similar to the increase in *Gfap* expression in the experimental eyes of non-Cre-expressing mice (*p* = 0.866, ANOVA).

**Figure 6:**
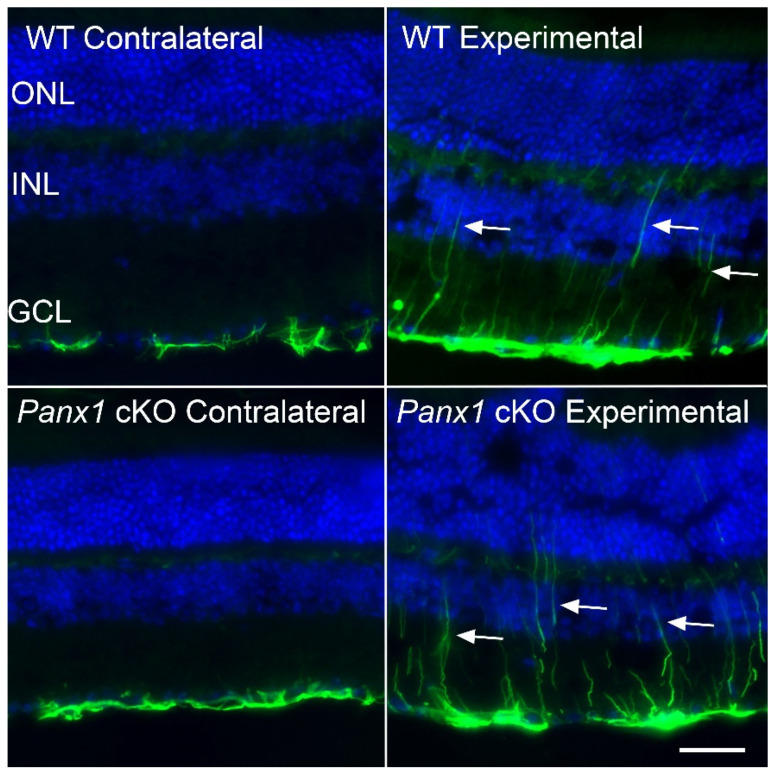
GFAP immunostaining of retinal sections. GFAP immunostaining of retinal sections from wild-type (WT) mice and mice with *Panx1* conditionally knocked out (cKO) in macroglia, showing both the contralateral and experimental retinas 7 days after optic nerve crush surgery. Contralateral retinas from both genotypes of mice exhibit GFAP localization restricted to the nerve fiber layer adjacent to the ganglion cell layer (GCL), indicative of staining in astrocytes. This pattern of staining was indistinguishable from the pattern observed in naïve retinas [19]. The experimental retinas exhibit GFAP staining in both the astrocytes and in Müller cells, which extend processes into the outer retina (arrows) and extend to the nerve fiber layer. Scale bar = 100 µm. Outer nuclear layer (ONL). Inner nuclear layer (INL).

Immunostaining for GFAP in frozen sections of retinas from mice at 7 days after optic nerve damage was performed to assess the cell types that exhibited expression of this intermediate filament. In wild-type mice, GFAP immunoreactivity was detected in both astrocytes and Müller cell processes in experimental retinas but was restricted to astrocytes in the contralateral eyes (Figure 6). Similarly, in the mice with conditional knock-out of *Panx1* in macroglial cells, both astrocytes and Müller cell processes were robustly stained for GFAP in experimental retinas. In contralateral retinas, however, we rarely detected Müller cell processes that were positive for GFAP, indicating that the majority of GFAP up-regulation in these retinas was restricted to the astrocyte population. These results indicate that cell-signaling among macroglial PANX1 channels is important for suppressing reactivity of astrocytes in retinas not immediately affected by optic nerve damage.

## 3. Discussion

Retinal astrogliosis in response to optic nerve injury is a continuum between astrocytic responses to promote repair to a state that effectively contributes to overall retinal pathology [8]. There is compelling evidence that reactive astrocytes contribute to RGC death in retinas directly affected by acute optic nerve damage [31] but the consequence of glial cell reactivity in contralateral retinas is not well understood.

In wild-type mice the level of astroglial reactivity, as a function of *Gfap* up-regulation, is suppressed in the contralateral retina, which may be a mechanism to reduce the risk of extending pathology to distant regions of the central nervous system. The data we present here suggest that part of this suppression is regulated by the activity of PANX1 channels. While the expression levels of GFAP have proportionately correlated to neuronal damage [56], it is not the definitive marker of pathologic gliosis. It remains to be determined if the astrocytes in the contralateral retinas of mice with *Panx1* deleted in the macroglia are in the A1 state of reactivity and if the RGCs are adversely affected. Preliminary evaluation of RGC density in contralateral retinas between wild-type and *Panx1^fl/fl^* animals suggests no difference in cell density even 21 days after unilateral optic nerve injury (wild type = 9.98 ± 0.79 cells/10^4^ µm^2^ vs. 9.78 ± 0.47 cells/10^4^ µm^2^, *p* = 0.91, *t*-test). However, undamaged RGCs, in vivo, are refractory to the toxic milieu of reactive astrocytes but are less resilient to a damaging stimulus presented by optic nerve damage [31]. Therefore, RGCs in the contralateral retina may experience a period where they have decreased resilience to damaging stimuli. Alternatively, 21 days may be an insufficient time point to accurately evaluate the pathology of RGCs in the contralateral retina. Studies indicate that unilateral crush injury can lead to moderate RGC loss (approximately 15%) in the contralateral retina by 45 days in mice where the optic nerve was damaged close to the globe [28,57]. This model of optic nerve damage is similar to the one reported here.

The role of PANX1 in suppressing over-expression of *Gfap* is contradictory to much of the literature, which depicts the PANX1-P2X7 receptor axis as instrumental in activating glial responses in the central nervous system [58,59,60,61]. These observations likely reflect an autocrine mechanism that promotes continued activation of the P2X7 channel. The role of PANX1 in regulating P2X7 is more complex, however, and several studies indicate that PANX1 can mediate permeability of P2X7, including modulating Ca^2+^ influx and channel conductance [42,44,46,62]. Such a role for PANX1 implies a negative regulatory autocrine signaling mechanism in astrocytes. Alternatively, a paracrine signaling mechanism could be considered since our *Panx1* conditional knock-out mice would also be expected to have this gene deleted in at least a subset of Müller cells. This is consistent with previous observations where *Panx1* was deleted in RGCs using AAV2 virus to deliver Cre recombinase in mice with the floxed *Panx1* allele. In these mice, macrogliosis was not inhibited but failed to subside over time after optic nerve crush [19]. While AAV2 has a high affinity for RGCs, it also transduces subsets of Müller cells, especially after retinal damage [63]. Further studies are required to elucidate the potential roles of both autocrine and paracrine signaling, and to delineate what effector molecule(s), are released through functional PANX1 hemichannels.

**Figure 7:**
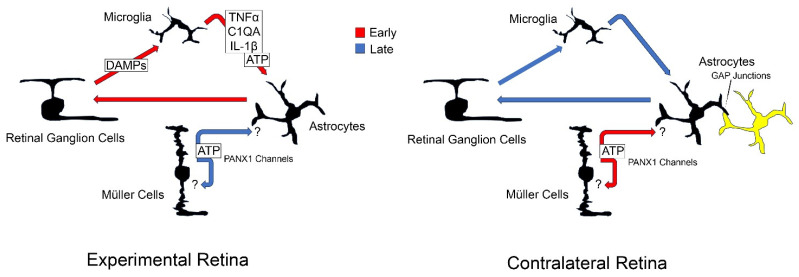
A hypothetical model of gliotic responses in the experimental and contralateral retinas after optic nerve damage. This model assumes the interplay of 4 retinal cell types that interact to both induce reactivity and suppress it. In the experimental retina (the retina directly affected by optic nerve damage), reactive gliosis is principally initiated by damage to the retinal ganglion cells (RGCs). This leads to the astrocyte response which is mediated through reactive microglia. Reactive astrocytes then present an unknown toxic signaling environment that mediates further RGC damage and loss leading to a continuous loop of pathology. The cycle is finally broken by an unknown purinergic signaling pathway that involves the release of ATP from PANX1 channels. Indirect evidence suggests this process is mediated by Müller cells and it is unknown if the signaling is autocrine or paracrine, or both (designated by the question marks). In the contralateral retina, astrocyte reactivity is mediated not by initial RGC pathology but by signaling from an astrocyte network (yellow cells) that extends from the site of injury through to the contralateral optic nerve and is facilitated by gap junctions between these cells. We predict that the purinergic signaling pathway that suppresses astrocyte reactivity in the experimental retina is activated early in the contralateral retina to help minimize activation of the pathology loop between RGCs, microglia, and astrocytes.

We envision a model of astrogliosis that has distinct pathways leading to astrocyte reactivity (Figure 7). In retinas directly affected by optic nerve damage, reactive gliosis is principally mediated by early RGC death [19]. Dying cells release damage-associated molecular pattern molecules (i.e., mitochondrial DNA as an example) that initiate microglial reactivity, which in turn leads to a reactive phenotype in astrocytes [30]. Studies using both agonists and antagonists of purinergic receptors suggests that one of the signaling ligands released by microglia is ATP. Reactive astrocytes then create a toxic environment that acts upon surviving RGCs [31] leading to a continuous loop of pathology in the retina. Studies using *Panx1*-conditional knock-out mice, including this one, suggests that eventually the reactive cycle of pathology is mitigated by additional activation of the purinergic signaling pathway, possibly from Müller cells, although whether or not this late event is an autocrine or paracrine mechanism is not known. We predict that the signaling events in the contralateral retina are quite different, both from the standpoint of astrocyte reactivity and the timing of signaling. Rather than responding to direct RGC pathology, astrocytes are receiving input from other astrocytes through a syncytium of cells extending all the way back to the site of damage. Studies suggest that the syncytium is interconnected through gap junctions [25,26,27]. Rather than being a late event, however, purinergic signaling that facilitates late reduction in astrogliosis in the experimental retina is activated early in the contralateral retina to help suppress this response, possibly as a mechanism to limit RGC pathology. Importantly, this is a hypothetical model and is intended to present a framework for future experimental testing.

## 4. Materials and Methods

### 4.1. Animals and the Optic Nerve Crush Procedure

Adult (3–5 months old) mice were handled in accordance with the Association for Research in Vision and Ophthalmology statement on the use of animals in research. All experimental protocols and the ethical care of the mice were reviewed and approved by the Institutional Animal Care and Use Committee of the University of Wisconsin. Mice were housed in microisolator cages and kept on a 12 h light/dark cycle and maintained on a 4% fat diet (8604 M/R; Harland Teklad, Madison, WI, USA). Mice harboring LoxP sites flanking exons 3 and 4 in a single-copy *Panx1* gene (*Panx1^fl^*^/*fl*^), as previously described [64], were used to generate *Panx1* macroglial conditional knockouts. *Panx1^fl/fl^* or *Rosa26*-(LoxP)-tdTomato (B6.Cg-Gt(ROSA)26Sor^tm9(CAG-tdTomato)Hze^, Jackson Laboratories, Bar Harbor, ME, USA) animals were crossed with transgenic mice carrying CRE recombinase under the control of mouse *Gfap* promoter (B6.C6-Tg(*Gfap*-cre)77.6Mvs/2J, Jackson Laboratories). All genotypes were on the C57BL/6 background and all studied cohorts contained an equal distribution of male and female mice.

Optic nerve crush was performed as previously described [65]. Briefly, mice were anesthetized with ketamine (120 mg/kg) and xylazine (11.3 mg/kg) and the eye numbed with a drop of 0.5% proparacaine hydrochloride (Akorn, Lake Forest, IL, USA). A lateral canthotomy was performed followed by an incision through the conjunctiva at the limbal junction, and the optic nerve was exposed and clamped for 5 s using self-closing N7 forceps (Roboz Surgical Instruments, Gaithersburg, MD, USA). After surgery, the eye was covered with triple antibiotic ointment and a subcutaneous injection of buprenex (0.2 mg/kg) was delivered to prophylactically alleviate pain. Surgery was not performed on the right eye of each mouse.

### 4.2. Immunostaining of Retinal Whole Mounts and Sections

At set times after optic nerve crush surgery (1, 3, 7, 14, and 21 days) mice were euthanized with a lethal overdose of pentobarbital sodium. A micro cautery was used to mark the position of the superior region of the retina before enucleation of the eye. After enucleation, globes were punctured with a 30 G needle and emersed in 4% paraformaldehyde in phosphate-buffered saline (PBS; 100 mM phosphate buffer, pH 7.2, and 150 mM NaCl) for 1 h at 37 °C followed by emersion in 0.4% paraformaldehyde in PBS and stored at 4 °C until all the samples were collected. The globes were then dissected into eye cups by removal of the anterior chamber and lens. The surface of the retina was carefully wiped with a Kimwipe twisted into a spear to remove traces of remaining vitreous humor. Eye cups were then equilibrated in 30% sucrose in PBS overnight at 4 °C.

For sections, equilibrated eye cups were embedded in Tissue-Plus Optimal Cutting Temperature (OCT) media (ThermoFisher Scientific, Waltham, MA, USA) and cut along an axis containing the optic nerve head at a thickness of 5 µm. Sections were adhered to glass Superfrost Plus slides (ThermoFisher Scientific). Slides were rinsed in PBS and then blocked in 0.1% Triton-X and 2% BSA in PBS overnight at 4 °C. The sections were then overlaid with either a rabbit polyclonal antibody against GFAP (1:1000 dilution in blocking buffer, Agilent Dako, Santa Clara, CA, USA, Cat # Z0334), a rabbit polyclonal antibody against SOX9 (1:1000 dilution, MilliporeSigma, Burlington, MA, USA, Cat # AB5535), or mouse monoclonal antibody against BRN3A (1:50 dilution, MilliporeSigma, Cat # MAB1585) and incubated in a humidified chamber overnight at 4 °C. After washing in PBS, the sections were then incubated with goat anti-rabbit or goat anti-mouse IgGs conjugated to FITC (1:1000 dilution, Jackson ImmunoResearch Inc., West Grove, PA, USA) for a minimum of 2 h. Slides were then thoroughly rinsed in PBS before being incubated with 300 ng/mL 4′,6-diamidino-2-phenylindole (DAPI) for 5 min at room temperature. Finally, the slides were rinsed in PBS and cover slipped with Immu-Mount (ThermoFisher Scientific) and stored at 4 °C in the dark.

For whole-mount staining, equilibrated eye cups in glass vials were subjected to 3 rounds of freezing and thawing on dry ice, washed with PBS and then incubated in blocking buffer (see above) overnight at 4 °C. Retinas were stained as eye cups using principally the same procedure as sections with the exception that staining with the primary antibody against GFAP was conducted for 3–4 days at 4 °C. Eye cups were then washed in PBS, incubated in second antibody overnight at 4 °C, and again washed in PBS. The secondary antibody used for whole mounts was a goat anti-rabbit IgG conjugated to Alexa488 (1:1500 dilution, Jackson ImmunoResearch Inc.). During the whole staining procedure, the superior pole of each eye cup and retina was identified with a single relaxing cut. The retinas were then dissected from the eye cup and mounted on glass Superfrost Plus slides using 3 additional relaxing cuts. After being briefly dried, the flattened retinas were mounted with Vectashield containing DAPI (Vector Laboratories, Burlingame, CA, USA) and cover slipped. Digital imaging of both stained sections and whole mounts was taken using a Zeiss AxioImager.Z2 epifluorescent microscope (Carl Zeiss Microscopy LLC, White Plains, NY, USA).

### 4.3. Astrocyte Morphology Scoring and Assessment of Retinal Coverage

Whole mounted retinas were all divided into roughly 4 equal lobes representing the superior/nasal, superior/temporal, inferior/nasal, and inferior/temporal quadrants of the retina. Each lobe was imaged in six locations (2 peripheral, 2 mid-peripheral, and 2 central fields) using a 20X air objective lens. All digital photography was made using a fixed exposure time and laser intensity. A minimum of 5 retinas were imaged at each time point after optic nerve crush surgery. For measurements of astrocyte coverage of the retina, all images were imported into ImageJ (v1.53k, National Institutes of Health, Bethesda, MD, USA), converted to greyscale, and then thresholded to highlight only the GFAP staining. Coverage was calculated as the area covered by the thresholded, stained area as a function of the area of the entire field.

**Figure 8:**
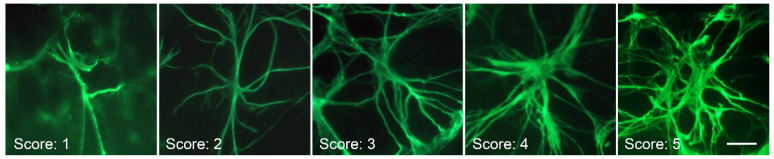
Exemplar of astrocyte hypertrophy scoring. Individual GFAP-immunostained retinal astrocytes showing increasing hypertrophy based on morphology. A score of 1 reflected cells with a principally bipolar shape with 2–4 major thin branches and minimal connection to other cells. A score of 2 reflected cells with 4 or more thin branches, also with minimal connections to other cells. A score of 3 indicated cells with an enlarged soma and multiple primary and secondary branches. A score of 4 indicated cells with thick somas and branches. The branches also exhibited multiple secondary, tertiary, and quaternary branches and numerous connections with adjacent astrocytes. A score of 5 indicated cells with numerous fan-shaped branches, including multiple connections. Scale bar = 40 µm.

To score astrocyte hypertrophy we first created an exemplar of cells at 5 different stages of reactivity (Figure 8). Images from the superior/nasal quadrant of photographed retinas were then used for scoring among 3 masked observers (JAW, JAD, RWN). All the astrocytes in each image were scored and compiled. Disagreements in scoring were defaulted to the majority agreement value.

### 4.4. RNA Isolation and Quantitative Analysis of mRNA Expression by Quantitative PCR

RNA isolations were performed as previously described [66]. Briefly, mice were euthanized with a lethal overdose of pentobarbital sodium, and retinal tissue was collected and flash-frozen on dry ice. Total RNA was isolated from a single retina, and 4 µg were treated with DNase I (Promega, Madison, WI, USA), purified by phenol/chloroform extraction, and converted to cDNA with oligo(dT) 15 primers and Moloney murine leukemia virus (M-MLV) reverse transcriptase (Promega). Samples of cDNA equivalent to 2 ng of mRNA were analyzed by quantitative PCR (qPCR) for changes in transcript abundance of *Panx1*, *Gfap*, and *S16* ribosomal protein. The cDNA was added to SYBR Green PCR master mix (Applied Biosystems, Grand Island, NY, USA) containing 0.25 μM of each primer in a 20 μL reaction volume. The primer sequences were: *Gfap*, F-5′CAAACTGGCTGATGTCACC and R-5′AGAACTGGATCTCCTCATCC; *Panx1*, F-5′GCTGCACAAGTTCTTCCCCT and R-5′ATCTCGAGCACTCTTGGCAG; *S16*, F-5′CACTGCAAACGGGGAAATGG and R-5′TGAGATGGACTGTCGGATGG. Cycling conditions were 95 °C (15 s) and 60 °C (60 s) for 40 cycles with a dissociation step. All PCR products were subcloned and confirmed by sequence analysis. Each cDNA sample was run in triplicate on an ABI 7300 Real Time PCR system (Applied Biosystems) and absolute transcript abundance was determined using a standard curve of the target molecule run on the same array. Data from different samples were normalized to S16.

### 4.5. Statistical Analysis

All data was plotted with individual samples (retinas) shown as individual points in a scatter plot along with the mean and standard deviation error bars. Astrocyte hypertrophy scores were plotted as a Box and Whisker plot showing the median score and error bars for the 10 and 90th percentiles. Comparisons of two individual cohorts of data were made using a Student’s two-sample *t*-test of the mean assuming equal variance. Comparisons of more than two cohorts of data were made using an ordinary one-way ANOVA. Statistical significance (alpha) was set as *p* ≤ 0.05.

## 5. Conclusions

Reactive astrogliosis is a feature of both experimental and contralateral retinas in a mouse model of unilateral optic nerve damage. When assessing *Gfap* gene expression as a marker for the astrocyte response, contralateral retinas showed a significant increase in the expression of this gene relative to naïve retinas but this was suppressed 4-fold relative to retinas directly connected to the damaged optic nerve. This suppression was negated in contralateral retinas, and restricted to astrocytes, in the absence of a functional PANX1 channel in macroglial cells. This suggests a novel negative feedback mechanism that prevents astrogliosis in regions of the central nervous system that are distant from the site of injury.

## Data Availability

Data will be made available by the authors upon reasonable request.
